# Antimicrobial and Cytotoxic Activities of Water-Soluble Isoxazole-Linked 1,3,4-Oxadiazole with Delocalized Charge: In Vitro and In Vivo Results

**DOI:** 10.3390/ijms242216033

**Published:** 2023-11-07

**Authors:** Bartłomiej Dudek, Urszula Bąchor, Ewa Drozd-Szczygieł, Malwina Brożyna, Piotr Dąbrowski, Adam Junka, Marcin Mączyński

**Affiliations:** 1 Platform for Unique Models Application (P.U.M.A), Department of Pharmaceutical Microbiology and Parasitology, Faculty of Pharmacy, Wroclaw Medical University, Borowska 211, 50-556 Wroclaw, Poland; bartlomiej.dudek@umw.edu.pl (B.D.); malwina.brozyna@student.umw.edu.pl (M.B.); 2 Department of Organic Chemistry and Drug Technology, Faculty of Pharmacy, Wroclaw Medical University, 211A Borowska Street, 50-556 Wroclaw, Poland; urszula.bachor@umw.edu.pl (U.B.); ewa.drozd-szczygiel@umw.edu.pl (E.D.-S.); marcin.maczynski@umw.edu.pl (M.M.); 3 Medical Department, Lazarski University, 02-662 Warsaw, Poland; piotr.dabrowski@lazarski.pl

**Keywords:** cationic organic compounds, antimicrobial activity, cytotoxicity, *Galleria mellonella* in vivo model

## Abstract

The distinct structure of cationic organic compounds plays a pivotal role in enhancing their water solubility, which in turn influences their bioavailability. A representative of these compounds, which contains a delocalized charge, is 5-amino-2-(5-amino-3-methyl-1,2-oxazol-4-yl)-3-methyl-2,3-dihydro-1,3,4-oxadiazol-2-ylium bromide (ED). The high-water solubility of ED obviates the need for potentially harmful solvents during in vitro testing. The antibacterial and antifungal activities of the ED compound were assessed in vitro using the microtiter plate method and a biocellulose-based biofilm model. Additionally, its cytotoxic effects on wound bed fibroblasts and keratinocytes were examined. The antistaphylococcal activity of ED was also evaluated using an in vivo larvae model of *Galleria mellonella*. Results indicated that ED was more effective against Gram-positive bacteria than Gram-negative ones, exhibiting bactericidal properties. Furthermore, ED demonstrated greater efficacy against biofilms formed by Gram-positive bacteria. At bactericidal concentrations, ED was non-cytotoxic to fibroblasts and keratinocytes. In in vivo tests, ED was non-toxic to the larvae. When co-injected with a high load of *S. aureus*, it reduced the average larval mortality by approximately 40%. These findings suggest that ED holds promise for further evaluation as a potential treatment for biofilm-based wound infections, especially those caused by Gram-positive pathogens like *S. aureus*.

## 1. Introduction

The increasing need for new antimicrobial and antibiofilm agents has intensified research into potent molecules to combat infections [1]. Many small molecules that contain heterocyclic elements are of pharmaceutical application/potential thanks to their broad biological activity [2]. Heterocyclic compounds are rapidly increasing in number due to extensive synthetic research and their synthetic utility [3] and play diverse roles in pharmacy and medicine, including anti-cancer, antimicrobial (including anti-biofilm), and anti-inflammatory actions, and they are a major focus in the organic chemistry literature [4,5,6].

Heterocyclic compounds have one or more ring structures, often with five or six atoms, and include atoms like nitrogen (N), oxygen (O), or sulfur (S). Specifically, heterocycles with nitrogen and oxygen are crucial in studies on natural products and drug synthesis [1]. The prominent examples of such compounds are isoxazoles and 1,3,4-oxadiazoles. Their therapeutic potential is growing, with oxadiazole derivatives being notable for their unique –N=C=O- linkage in medicinal chemistry [7,8,9]. As an example, drugs such as Furamizol and Zibotentan, which contain the 1,3,4-oxadiazole ring, are applied clinically thanks to their proven therapeutic value [10,11].

The further development of new heterocyclic compounds with beneficial biological effects is vital for advancing medicinal chemistry and drug discovery. Designing effective drugs involves modifying their chemical and physical properties to improve solubility, decrease toxicity, and optimize other pharmacokinetic properties. A drug molecule’s water solubility, influenced by factors like the partition coefficient and localized charge, affects its absorption. Contrary, molecules with limited solubility can have absorption issues [12].

Building on our prior work on isoxazoles [13], in this work we investigated the biological properties of the water-soluble compound: 5-amino-2-(5-amino-3-methyl-1,2-oxazol-4-yl)-3-methyl-2,3-dihydro-1,3,4-oxadiazol-2-ylium bromide, referred to in this work as ED. Our primary focus was to determine whether this compound could be considered a potential (initial) candidate for addressing biofilm-related chronic wound infections. To answer this question, we conducted a dual investigation: first, we evaluated in vitro ED’s antimicrobial/antibiofilm capabilities; subsequently, we studied its cytotoxic effects on wound bed fibroblasts and keratinocytes. We then combined the results from these in vitro studies to assess both the antibacterial effectiveness and cytotoxicity of ED in vivo, using the *Galleria mellonella* larvae as a model for living, infected tissue. Our primary goal was thus to understand the potential of ED, emphasizing its possible role in treating biofilm-affected chronic wounds.

## 2. Results

**Figure 1:**
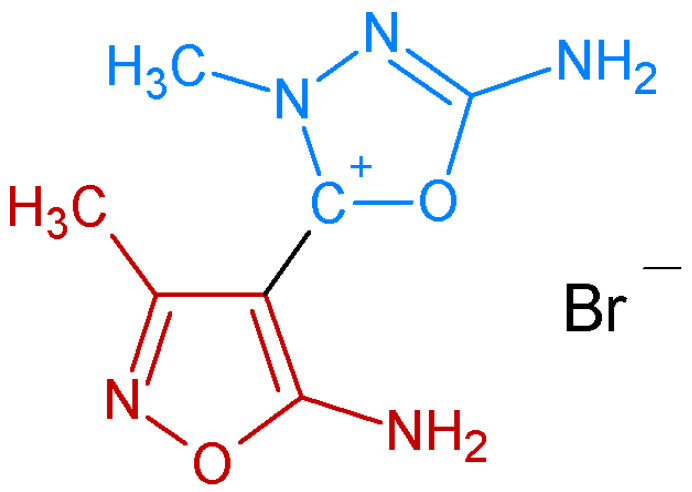
The chemical structure of investigated 5-amino-2-(5-amino-3-methyl-1,2-oxazol-4-yl)-3-methyl-2,3-dihydro-1,3,4-oxadiazol-2-ylium bromide (ED). The isoxasazole ring is marked red, while oxadiazole ring is marked blue color.

The synthesis, spectroscopic and structural analyses, as well as computational studies of ED molecule, have been previously delineated by our team [14], while its chemical structure is presented in Figure 1.

In initial microbiological tests, we measured the minimal inhibitory concentrations (MIC values) of ED using the microtiter plate method. We tested it against reference strains of Gram-positive and Gram-negative bacteria, as well as yeast-like fungus. The results showed that the ED compound had antimicrobial activity (MIC) at the concentrations tested (Table 1).

**Figure 2:**
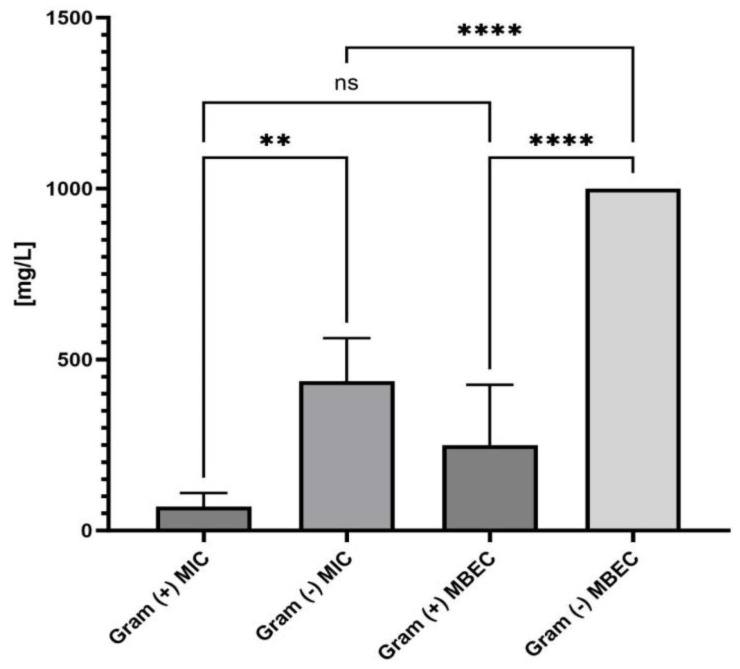
Comparison of MIC and MBEC values of analyzed compound towards all Gram (+) vs. Gram (−) bacteria. Asterisks show statistical significance, while “ns” denotes lack of statistical significance. The asterisks are intended to flag levels of significance. ANOVA with Tukey’s multiple comparison test (*p* = 0.05).

Also, **ED** efficacy against Gram-positive bacteria was significantly higher than against Gram-negative bacteria (Figure 2).

**Figure 3:**
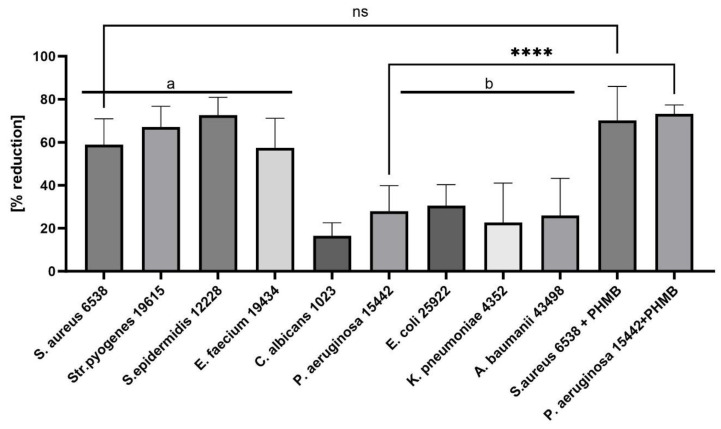
Comparison of ability of 500 mg/L **ED** compound to reduce biofilm [%] in the cellulose-based biofilm model (CBB). The asterisks or “ns” show statistical significance or insignificance, respectively, between level of reduction displayed by **ED** compound or standard PHMB antiseptic towards *S. aureus* and *P. aeruginosa* biofilm. The letters a/b show statistical significance in reduction between Gram+ and Gram− pathogens, while ns/**** shows lack or significant difference between reduction in biofilm performed by ED vs. PHMB, respectively. The asterisks are intended to flag levels of significance. ANOVA with Tukey’s multiple comparison test (*p* = 0.05).

Specifically, ED worked best against *S. epidermidis* (31.25 mg/L) and showed strong activity against *S. aureus* and *Str. pyogenes* (both at 62.5 mg/L). Noteworthy, the MBEC values towards Gram-positive bacteria were 2–4 times higher than the MIC/MBC values. This is consistent with other studies that have found biofilms to be more tolerant to antimicrobials than individual, free-floating bacteria in the planktonic state. In turn, for Gram-negative bacteria (except for *A. baumanii*), the MBEC value was beyond the tested range of concentrations. We subsequently examined the antibiofilm properties of the ED compound (Figure 3) using an alternative, biocellulose-based model. As a control to ensure the method’s validity, the PHMB (polyhexamethylene biguanide) antiseptic of proven activity was applied. As shown in Figure 3, ED was able to reduce biofilm also in the cellulose-based model, however, not completely, which stays in line with the data presented in Figure 2. Noteworthy, for Gram-positive wound pathogens, ED’s antiseptic effect was comparable to the reference, clinically applied antiseptic used in this study.

**Figure 4:**
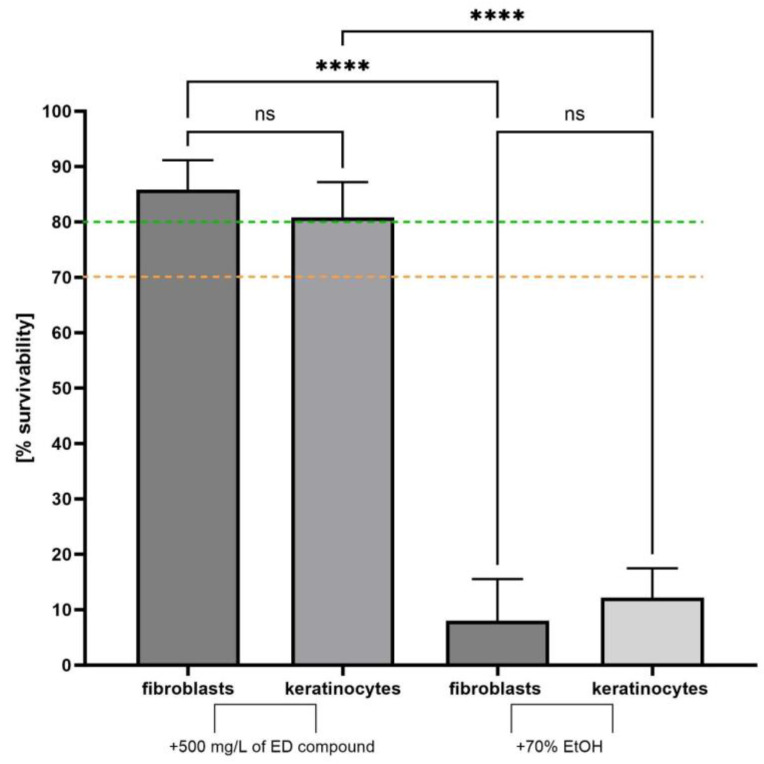
The survivability of fibroblast and keratinocytes cells exposed on the 500 mg/L of **ED** compound or 70% ethanol (usability control of experiment). The green dotted line indicates level of none or negligible cytotoxicity, while the orange dotted line indicates level of low/moderate cytotoxicity. Asterisks show statistical significance, while “ns” denotes lack of statistical significance. ANOVA with Tukey’s multiple comparison test (*p* = 0.05).

The ideal wound antiseptic should not only exhibit robust antimicrobial (antibiofilm) activity but also refrain from adversely affecting wound bed cells, such as fibroblasts and keratinocytes. Given the pivotal role of fibroblasts and keratinocytes in the wound healing process, we assessed their viability when exposed to the ED compound (Figure 4). For this evaluation, a concentration of 500 mg/L of ED was used.

This in vitro study revealed that ED did not exhibit cytotoxic effects on the cells forming the wound bed (fibroblasts and keratinocytes), in contrast to the effects observed when cells were treated with 70% ethanol (control setting). The obtained in vitro results led us to conduct a subsequent phase that bridged the previous two lines of research, i.e., to test the ED compound using a *Galleria mellonella* larvae. This animal test enables not only a fast and convenient way to assess the in vivo toxicity of a given compound (results show a strong correlation to those in mammalian systems) but also allows to check whether the applied antimicrobial is able to counteract spreading infection within the living organism containing pharmacological/pharmacokinetic compartments as well as the simple immune system.

**Figure 5:**
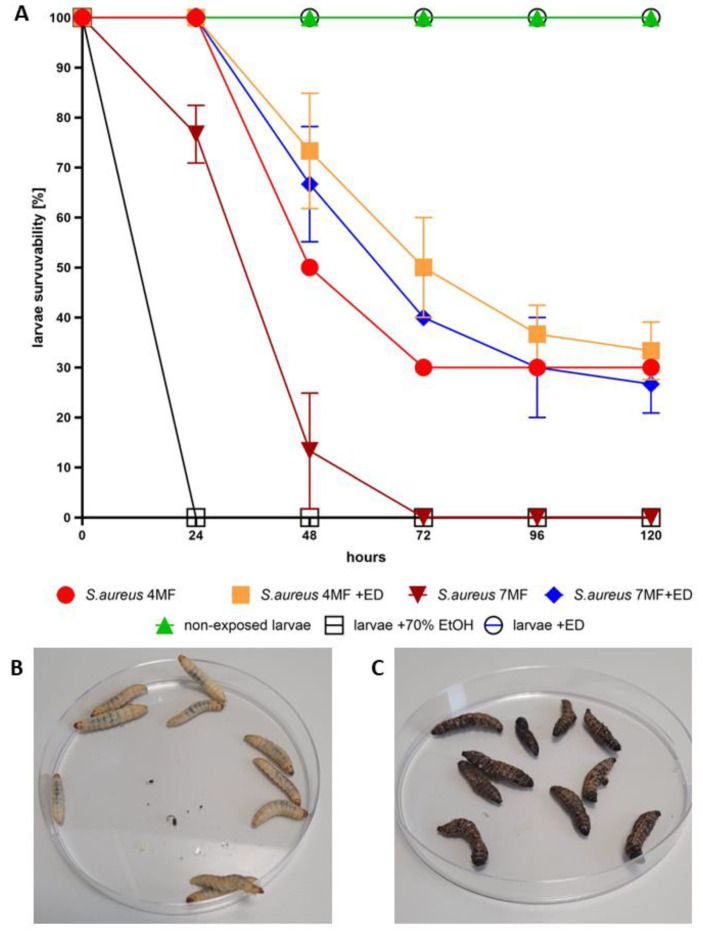
(**A**) The survivability [%] of larvae in the following hours (0–120) after exposure on the 500 mg/L of **ED** compound, or 70% ethanol (experiment usability control), or the PBS buffer (non-exposed larvae, control of growth). The infection model consisted of larvae injected with 4 or 7 McFarland (4 or 7MF, respectively) of *S. aureus* together with 500 mg of **ED** compound. The larvae injected with 4 or 7 MF of *S. aureus* only (without **ED** compound) served as the proof of this specific staphylococcal strain’s ability to kill larvae within time of experiment. (**B**) Live, creamy, motile larvae. (**C**) Dead, flaccid, motionless larvae.

The results presented in Figure 5 show that non-exposed larvae displayed full survivability within the timeframe of experiment (120 h), while larvae injected with toxic concentration of ethanol died within 24 h after exposure. Also, all larvae injected with 7 MF of *S. aureus* died within 72 h, while the average mortality of larvae injected with lower concentration of this pathogen (4 MF) reached 50% and 70% after 48 h and 72 h after exposure, respectively. The above-mentioned set of appropriate control results show the usability of *Galleria mellonella* to test the cytotoxicity and protective potential of ED in vivo against spreading staphylococcal infection. The provision of ED in the concentration of 500 mg/L to larvae infected with 7MF of staphylococcal cells decreased the average mortality of insects by 50% till the 72nd hour post injection and by ca. 40% after the 72nd hour. Noteworthy, at this time point, all larvae devoid of protective activity of ED and infected with 7 MF of staphylococci were already dead. This result confirms significant anti-staphylococcal activity of ED in the actual living tissue containing all obstacles typical for the living complex systems that may potentially diminish the antimicrobial potential of a given substance.

## 3. Discussion

Chronic wounds disrupt the continuity of skin and soft tissues, deviating from the typical healing trajectory [15]. A significant factor contributing to this perilous and often life-threatening phenomenon is the presence of microbial cells within the wound. These cells form a persistent structure known as a biofilm. This complex, multilayered community of microbial cells is highly resistant to both topically applied antiseptic agents and systemically administered antibiotics [16]. As a result, the search for novel antimicrobial agents—beyond traditional antibiotics and antiseptics—is crucial in the fight against wound biofilms. Additionally, any new antimicrobial agent must prioritize the safety of the patient’s cells essential for wound healing, such as fibroblasts and keratinocytes.

In this context, our paper investigates the antimicrobial/antibiofilm efficacy and cytotoxicity of the **ED** compound. This molecule, (5-amino-2-(5-amino-3-methyl-1,2-oxazol-4-yl)-3-methyl-2,3-dihydro-1,3,4-oxadiazol-2-ylium bromide), exemplifies an organic salt. Such salts consist of an organic cation paired with either inorganic or organic anions. These combinations yield unique properties, including interactions with biomolecules and other compounds [8]. The synthesis of the **ED** salt involved pairing the organic cation with Br^−^ as a counterion, resulting in a molecule with enhanced water solubility. This characteristic significantly affects its bioavailability.

In general, **ED** has shown antibacterial/antibiofilm and antifungal activity, with the most pronounced effect against Gram-positive bacteria (Table 1, Figure 2 and Figure 3). Importantly, it does not harm fibroblasts and keratinocytes, as evidenced in Figure 4. In an in vivo model, **ED** effectively prevented staphylococcal infection spread without causing harm to larvae (Figure 5). Nevertheless, several main pinpoints require further mentioning or discussion. These include:

(a) High Water Solubility: A clear advantage of the **ED** compound as an antimicrobial agent for chronic wounds is its high water solubility. This facilitates in vitro testing by eliminating the need for potentially harmful solvents. Moreover, water-soluble compounds are generally more suitable for medical applications, reducing risks and enhancing patient safety [17];
(b) Antimicrobial Activity: As indicated in Table 1, the MIC values of **ED** matched the MBC values, suggesting that **ED** not only inhibits but also kills the tested microorganisms. Such antimicrobial activity, as opposed to a bacteriostatic mode of action, is essential for topical treatment of wound infections [18];
(c) Gram-Positive Susceptibility: Gram-positive pathogens were more susceptible to the **ED** agent than Gram-negative bacteria and fungi. The differential susceptibility between these bacterial groups to antimicrobial agents can be attributed to several structural and functional differences, including cell wall composition, outer membrane presence, efflux pumps, target accessibility, and intrinsic resistance mechanisms [19,20]. To pinpoint **ED**’s exact mechanism of action, further studies are undoubtedly required. These should encompass various lines of investigation, such as assessing **ED**’s interaction with bacterial membranes using techniques like transmission electron microscopy, determining the compound’s uptake and efflux in both bacterial types, and identifying the precise target or mode of action. Understanding this mechanism can offer insights into optimizing the **ED** compound or devising adjuvant strategies to boost its activity against Gram-negative pathogens. Previous studies have indicated that interactions with charged compounds differ between these bacterial groups [21,22]. For instance, Abebe et al. synthesized a series of decyl-o-phenanthrolinium organic salts and found that the type of anion significantly influenced their antibacterial activities [23]. Other compounds with broad antimicrobial activity, such as cationic antimicrobial peptides (AMPs), have been identified. These peptides, typically short and positively charged, exhibit activity against a range of Gram-positive bacteria. Although their exact mode of action remains elusive, it is proposed that these peptides interact with and disrupt the cytoplasmic membrane, leading to cell death. Friedrich et al. demonstrated the high activity of positively charged compounds against Gram-positive strains of *S. aureus* [24]. Fletcher et al. observed that 1,3,4-trisubstituted-1,2,3-triazolium bromide salts had the lowest MIC values against Gram-positive bacteria [25]. Our spectroscopic analysis of the **ED** structure suggests that the presence of a positive charge is crucial for its selective antibacterial activity, as well as the presence of the hydrophobic isoxazole ring. To explain the non-specific activity of the ED compound, it is worth analyzing reports on positively charged structures, their antibacterial effects, and known mechanisms of action. Da Costa et al. [26] investigated the impact of surface charge modulation of rifampicin-loaded PLA (poly-lactic acid) nanoparticles (NPs) on behavior within a bacterial biofilm. The nanoparticles were functionalized with poly-L-lysine to achieve a positively charged surface. The observed effect was that positively charged nanoparticles had a stronger interaction with *S. aureus*, under biofilm growth conditions, than negatively charged ones. This led to slower particle migration in the biofilm and better retention of these cationic particles within the biofilms. Although rifampicin showed complete ineffectiveness in biofilms after washing, rifampicin-loaded PLA nanoparticles coated with poly-L-lysine exhibited enhanced antibiotic efficacy compared to uncoated, negatively charged nanoparticles. While negatively charged PLA NPs migrated rapidly and uniformly through the entire *S. aureus* biofilm, positively charged NPs were primarily bound to the top of biofilms and entrances of dense bacterial clusters, preventing deeper penetration. This suggests electrostatic interactions between the positive charge of the compound and the negatively charged biofilm components, such as bacteria and matrix macromolecules like eDNA. Hasan et al. demonstrated that cationic PLGA (Poly-lactic-co-glycolic acid) nanoparticles loaded with clindamycin significantly reduced the bacterial load in wounds of *S. aureus*-infected mice compared to anionic NPs [27]. This finding correlated with better wound healing, evidenced by a significantly smaller wound size after 8 days of local treatment. Coating nanoparticles with a poly-L-lysine corona inverted the surface charge of anionic NP-rifampicin, producing positively charged NP-rifampicin that exhibited higher interactions with planktonic S. aureus and biofilms than negatively charged NPs. This study revealed the importance of the ability of antibiotic-loaded NPs to penetrate and remain within biofilms for effective control. The stronger interactions observed with positively charged nanoparticles resulted in better retention of antibiotic-loaded nanoparticles within biofilms, enabling the sustainable delivery of potent rifampicin concentrations at reduced doses. These findings suggest that the carrier retention capacity within biofilms correlates with treatment effectiveness. Thus, positively charged rifampicin-loaded PLA nanoparticles present a promising approach for enhancing antibiotic delivery In *S. aureus* biofilms. Romero-Urbina et al. [28] reached conclusions aligned with the above due to the presence of a positive charge in the compound. They studied the effects of positively charged silver nanoparticles against both methicillin-sensitive and methicillin-resistant *S. aureus*. They examined the bactericidal effects of silver nanoparticles and the induced bacterial ultrastructural modifications using aberration-corrected transmission electron microscopy. The study showed that silver nanoparticles cause thinning and increased permeability of the cell wall, destabilization of the peptidoglycan layer, and consequent leakage of intracellular content, leading to bacterial cell lysis. They hypothesized that positively charged silver nanoparticles primarily bind to the negatively charged polyanionic backbones of teichoic acids and related bacterial cell wall glycopolymers, thereby affecting cell wall structure and permeability. This hypothesis could explain the antibacterial effects of silver nanoparticles on *S. aureus*.

In summary, while the exact mechanism of **ED**’s action remains unknown, findings from other researchers seem to corroborate our data.

(d) In Vivo *Model: G. mellonella* larvae are a widely used in vivo invertebrate infection model for studying bacterial and fungal infections and evaluating the efficacy of antimicrobial drugs [29]. We utilized *G. mellonella* larvae to assess the antibacterial efficacy and cytotoxicity of **ED** [30]. The injection of ED into the larvae significantly hindered *S. aureus’s* ability to cause a lethal infection (Figure 5). Notably, our experiment aimed to mimic the introduction of an antimicrobial agent to a chronic wound. The conditions within *G. mellonella* tissues, such as specific temperature, hypoxia, and the presence of immune system components, pose challenges not only for the antimicrobial agent due to pharmacokinetic/dynamic issues [31] but also for *S. aureus* biofilm development compared to standard in vitro models [32]. These conditions resemble those in necrotic, exuding chronic wounds [33].

Given that **ED** exhibited no cytotoxicity in our in vivo model and simultaneously protected the model organism from infection spread, further studies on **ED**, including its mode of action and efficacy in other animal models, are warranted. This is a crucial step towards introducing new, safe antimicrobial drugs capable of counteracting microbial infections.

## 4. Materials and Methods

### 4.1. Determination of Minimal Inhibitory Concentration Using Microtiter Plate Method

To ascertain the lowest concentration at which the examined compounds inhibit microbial growth (MIC values), the subsequent procedure was adopted. Initially, the strains were cultured overnight in TSB medium (Tryptic Soy Broth, Biomaxima, Lublin, Poland). Following this, 100 µL of TBS was introduced into every well of the 96-well plate, with the first column receiving the initial aliquot. This was followed by tenfold serial dilutions for each compound. A 0.5 MacFarland standard of bacterial/fungal suspensions was then achieved in a 0.9% sodium chloride solution (NaCl, Stanlab, Lublin, Poland) utilizing a densitometer (Densilameter II Erba Lachema, Brno, Czech Republic). These suspensions underwent a 1000-fold dilution in TSB, and 100 µL was dispensed into the wells containing the compounds. Controls for microbial growth (suspensions in TSB) and medium sterility (medium alone) were set up. The solution’s absorbance was gauged at 580 nm with a spectrophotometer (Multiskan Go, Thermo Fisher Scientific, Vantaa, Finland). Subsequently, the plates were placed in an incubator at 37 °C for 24 h, agitated at 350 rpm (Mini-shaker PSU-2T, Biosan SIA, Riga, Latvia). Post-incubation, the absorbance was again measured at 580 nm. To pinpoint the MIC values, 20 µL of 1% (*w*/*v*) TTC (2,3,5-triphenyl-tetrazolium chloride, AppliChem Gmbh, Darmstadt, Germany) in TSB was dispensed into every well, followed by a 2-h incubation under identical conditions. The MIC value for bacteria was identified in the initial well where no red hue was evident. Each compound underwent this process thrice. The entirety of the first well’s content (100 µL) that did not exhibit a red shift was relocated to 10 mL of the suitable broth and incubated for 72 h at the optimal temperature in a microbiological incubator. If no turbidity appeared in the broth within the 72-h window, the specific concentration of ED used was also deemed MBC. The strains exposed to sterile saline, instead of antimicrobial agent, served as the growth control setting.

### 4.2. Determination of Minimal Biofilm Eradication Concentration Using Microtiter Plate Model

The strains were cultured overnight using the Brain Heart Infusion medium. Following this, bacterial/fungal suspensions were standardized to a 0.5 MacFarland concentration using a 0.9% sodium chloride solution with the help of a densitometer. These suspensions underwent a 1000-fold dilution in TSB. A volume of 100 µL from this diluted suspension was then introduced into the wells of a 96-well plate and incubated at 37 °C for 18 h. Post-incubation, the medium was discarded, retaining only the cells that form biofilm. Thereafter, 100 µL of sterile broth was dispensed into each well. Geometric dilutions of the compounds under investigation were then carried out directly in the plate wells. This setup was incubated again at 37 °C for 18 h. After this period, 20 µL of 1% (*w*/*v*) TTC in TSB was added to every well, followed by a 2-h incubation under the same conditions. The MBEC value for bacteria was identified in the initial well that did not display a red hue. For *C. albicans*, 20 µL of 0.1% resazurin in TSB was added to each well and given a 3-h incubation under identical conditions. The MBEC value for *C. albicans* was determined in the first well where a blue shade was evident. This entire procedure was replicated thrice for every compound and its respective concentration. The equation employed to calculate biofilm eradication was 100% minus [absorbance value from biofilm treated with the compound in question divided by absorbance value from biofilm treated with saline) multiplied by 100%]. These tests were conducted in three iterations.

### 4.3. The Cytotoxicity Assay of Analyzed Compounds towards Fibroblast and Keratinocytes Cell Line In Vitro

The Neutral Red (NR) cytotoxicity test was conducted on fibroblast and keratinocytes (L929 and HaCaT, ATCC, Manassas, VA, USA) in vitro cell cultures. These cultures were exposed to the compounds under study at concentrations equivalent to their MBEC values (or MIC if the MBEC exceeded the tested concentration range) for 24 h. This was done in alignment with biological evaluation of medical devices; Part 5: In vitro cytotoxicity tests; Part 12: Sample preparation and reference materials. The compounds were dissolved in the cell culture medium (RPMI, Sigma-Aldrich, Darmstadt, Germany) and allowed to air-dry at ambient temperature. Subsequently, 150 µL of a de-staining solution (comprising 50% ethanol (96%), 49% deionized water, and 1% glacial acetic acid; sourced from POCH, Lublin, Poland) was added to each well. The plate was then vigorously agitated using a shaker (MTS4, IKA-Labortechnik, Berlin, Germany) for 30 min, ensuring the NR was fully extracted from the cells, resulting in a uniform solution. The absorbance of the NR was then spectrometrically gauged using a microplate reader at a wavelength of 540 nm. The absorbance value from NR-stained cells that were not exposed to the compound-infused medium was deemed as 100% potential cell growth (serving as the positive growth control). Conversely, cells exposed to 70% EtOH (from POCH, Lublin, Poland) for 30 min were used as the method’s control. Each analysis was replicated six times.

### 4.4. The Synthesis and Purification of Bacterial Cellulose Carrier

A suspension of 2 × 10^5^ CFU/mL of *Komagateibacter xylinus* DSM 46604, derived from a week-long culture, was added to a custom-prepared Herstin–Schramm medium. This medium contained ingredients such as: glucose (2% *w*/*v*; POCH, Gliwice, Poland), yeast extract (0.5% *w*/*v*; Graso, Starogard Gdanski, Poland), bacto-pepton (0.5% *w*/*v*; Graso, Starogard Gdanski, Poland), citric acid (0.115% *w*/*v*; POCH, Gliwice, Poland), Na_2_HPO_4_ (0.27% *w*/*v*; POCH, Gliwice, Poland), MgSO_4_ × 7H_2_O (0.05% *w*/*v*; POCH, Gliwice, Poland), bacteriological agar (2% *w*/*v*; Graso, Starogard Gdanski, Poland), and ethanol (1% *v*/*v*; POCH, Gliwice, Poland). The synthesis of bacterial cellulose took place in 24-well plates. The resulting bacterial cellulose carriers (BC), shaped as 18-mm diameter cylinders, underwent alkaline lysis to eliminate *K. xylinus* cells. They were then meticulously washed with sterile water until a neutral pH was achieved.

### 4.5. Assessment of ED Compound’s Antibiofilm Activity Using Cellulose-Based Biofilm Model

The proliferation of microbes on BC disks was gauged using a standard tetrazolium test, conducted every 24 h over a week. Subsequently, 2 mL of 10^5^ CFU/mL of the pathogens under study was applied to the BC. A 24-h culture of these microbes served as a model for ED application, as previously demonstrated [34]. The biofilm on the BC carrier was then exposed to 500 mg/L of the ED compound for an hour. Following this, the carriers with the residual biofilm were moved to 10 mL of a neutralizing solution (peptone saline water containing 1 g/L casein and 8.5 g/L NaCl) for 5 min. Post-neutralization, the BCs were added to 2 mL of BHI medium (Biomaxima, Lublin, Poland) infused with 1% TTC and incubated for 2 h. After this period, the medium was discarded, and the BCs were washed with 0.9% NaCl. Next, 1 mL of a solution (comprising ethanol: acetic acid in a 90:10 *v/v* ratio, sourced from POCH, Gliwice, Poland) was added to the BCs to extract formazan. This was followed by a 15-min mechanical agitation. The formazan solution was then transferred to new 96-well plates and its concentration was determined at 490 nm using the MultiScan Go Spectrophotometer (Thermo Fischer Scientific, Waltham, MA, USA). This was done to evaluate the percentage of biofilm-forming cells left on the BC samples relative to the untreated control. BCs with pre-established biofilm treated with 0.9% NaCl, instead of the ED compound, acted as the biofilm growth control. The biofilm eradication was computed by division of of the average absorbance values for the test samples per average absorbance values for the control samples.

### 4.6. Larvae In Vivo Model to Assess Compound’s Cytotoxicity and Capability to Prevent Infection

The in vivo model with *Galleria mellonella* larvae was performed to assess ED cytotoxicity and confirm the ED anti-staphylococcal activity in vivo. Sixth instar larvae of the greater wax moth, *G. mellonella*, with an average weight of 0.21 g, were selected for the experiment. The larvae were injected with 20 µL of ED solution (reaching 500 mg/L/body mass) to evaluate its cytotoxicity or with 10 µL of the *S. aureus* ATCC 6538 (American Type Culture Collection) strain at 1.25 × 10^9^ CFU/mL or with 10 µL of the *S. aureus* 6538 and 20 µL of ED simultaneously. The 4 or 7 MF density of microbial strain was applied. Moreover, a negative control with 10 µL of PBS (Dulbecco′s Phosphate Buffered Saline, Biowest, Riverside, MO, USA) and a usability control with 10 µL of 96% (*v*/*v*) ethanol (Stanlab, Lublin, Poland) were performed. The larvae were placed in 90-mm Petri dishes (Noex, Warsaw, Poland) and incubated at 30 °C for five days. Each day, the mortality of larvae was monitored. Death was defined when the larvae were nonmobile, melanized, and did not react to physical stimuli. Three groups of 10 larvae were analyzed for each testing condition.

### 4.7. The Statistical Analysis

Statistical analyses were performed using GraphPad Prism 8.0. (GraphPad 10 Software, San Diego, CA, USA). Normality of distribution was verified using Shapiro–Wilk’s test. To evaluate statistical significance, the ANOVA with Tukey’ post hoc multiple comparison test was performed with *p* < 0.05.

## Figures and Tables

**Table 1:** The values of Minimal Inhibitory Concentration (MIC), Minimal Biocidal Concentration (MBC) and Minimal Biofilm Eradication Concentration (MBEC) of analyzed **ED** compound against *Staphylococcus aureus*, *Streptococcus pyogenes*, *Staphylococcus epidermidis*, *Enterococcus faecium*, *Candida albicans*, *Pseudomonas aeruginosa*, *Escherichia coli*, *Klebsiella pneumoniae*, and *Acinetobacter baumanii*.

		MIC = MBC [mg/L]	MBEC
Gram (+) bacteria	*S. aureus* 6538	62.5	125
	*Str. pyogenes* 19615	62.5	250
	*S.epidermidis* 12228	31.25	125
	*E. faecium* 19434	125	500
fungus	*C.albicans* 1023	250 [Minimal Fungicidal Concentration]	1000
Gram (−) bacteria	*P.aeruginosa* 15442	500	>range of concentrations
	*E.coli* 25922	500	>range of concentrations
	*K.pneumoniae* 4352	500	>range of concentrations
	*A.baumanii* 43498	250	1000

## Data Availability

All data is presented in the manuscript. Any additional information will be shared on reasonable request to the corresponding author.

## References

[1] Shrestha L., Fan H.M., Tao H.R., Huang J.D. (2022). Recent Strategies to Combat Biofilms Using Antimicrobial Agents and Therapeutic Approaches. Pathogens.

[2] Sharma P.K., Amin A., Kumar M. (2020). Synthetic Methods of Medicinally Important Heterocyclesthiazines: A Review. Open J. Med. Chem..

[3] Quadir T., Amin A., Sharma P.K., Jeelani I., Abe H. (2022). A Review on Medicinally Important Heterocyclic Compounds. Open J. Med. Chem..

[4] Vaidya A., Pathak D., Shah K. (2021). 1,3,4-oxadiazole and its derivatives: A review on recent progress in anticancer activities. Chem. Biol. Drug Des..

[5] Qadir T., Kanth S.A., Aasif M., Fadul A.N., Yatoo G.N., Jangid K., Mir M.A., Shah W.A., Sharma P.K. (2023). Design, synthesis, and unraveling the antibacterial and antibiofilm potential of 2-azidobenzothiazoles: Insights from a comprehensive in vitro study. Front. Chem..

[6] Arya G.C., Kaur K., Jaitak V. (2021). Isoxazole derivatives as anticancer agent: A review on synthetic strategies, mechanism of action and SAR studies. Eur. J. Med. Chem..

[7] Siwach A., Verma P.K. (2020). Therapeutic potential of oxadiazole or furadiazole containing compounds. BMC Chem..

[8] Glomb T., Świątek P. (2021). Antimicrobial Activity of 1,3,4-Oxadiazole Derivatives. Int. J. Mol. Sci..

[9] Sysak A., Obmińska-Mrukowicz B. (2017). Isoxazole ring as a useful scaffold in a search for new therapeutic agents. Eur. J. Med. Chem..

[10] Janardhanan J., Chang M., Mobashery S. (2016). The oxadiazole antibacterials. Curr. Opin. Microbiol..

[11] Cheng K., Qi J., Ren X., Zhang J., Li H., Xiao H., Wang R., Liu Z., Meng L., Ma N. (2022). Developing Isoxazole as a Native Photo-Cross-Linker for Photoaffinity Labeling and Chemoproteomics. Angew. Chem. Int. Ed..

[12] Presentato A., Piacenza E., Scurria A., Albanese L., Zabini F., Meneguzzo F., Nuzzo D., Pagliaro M., Martino D.C., Alduina R. (2020). A New Water-Soluble Bactericidal Agent for the Treatment of Infections Caused by Gram-Positive and Gram-Negative Bacterial Strains. Antibiotics.

[13] Bąchor U., Junka A., Brożyna M., Mączyński M. (2023). The In Vitro Impact of Isoxazole Derivatives on Pathogenic Biofilm and Cytotoxicity of Fibroblast Cell Line. Int. J. Mol. Sci..

[14] Bąchor U., Drozd-Szczygieł E., Bąchor R., Jerzykiewicz L., Wieczorek R., Mączyński M. (2021). New water-soluble isoxazole-linked 1,3,4-oxadiazole derivative with delocalized positive charge. RSC Adv..

[15] Schilrreff P., Alexiev U. (2022). Chronic Inflammation in Non-Healing Skin Wounds and Promising Natural Bioactive Compounds Treatment. Int. J. Mol. Sci..

[16] Diban F., Di Lodovico S., Di Fermo P., D’Ercole S., D’Arcangelo S., Di Giulio M., Cellini L. (2023). Biofilms in Chronic Wound Infections: Innovative Antimicrobial Approaches Using the In Vitro Lubbock Chronic Wound Biofilm Model. Int. J. Mol. Sci..

[17] Budiman A., Rusdin A., Aulifa D.L. (2023). Current Techniques of Water Solubility Improvement for Antioxidant Compounds and Their Correlation with Its Activity: Molecular Pharmaceutics. Antioxidants.

[18] Negut I., Grumezescu V., Grumezescu A.M. (2018). Treatment Strategies for Infected Wounds. Molecules.

[19] Chetri S. (2023). The culmination of multidrug-resistant efflux pumps vs. meager antibiotic arsenal era: Urgent need for an improved new generation of EPIs. Front Microbiol..

[20] Mai-Prochnow A., Clauson M., Hong J., Murphy A.B. (2016). Gram positive and Gram negative bacteria differ in their sensitivity to cold plasma. Sci. Rep..

[21] Caudill E.R., Hernandez R.T., Johnson K.P., O’Rourke J.T., Zhu L., Haynes C.L., Feng Z.V., Pedersen J.A. (2020). Wall teichoic acids govern cationic gold nanoparticle interaction with Gram-positive bacterial cell walls. Chem. Sci..

[22] Malanovic N., Lohner K. (2016). Gram-positive bacterial cell envelopes: The impact on the activity of antimicrobial peptides. Biochim. Biophys. Acta.

[23] Abebe A., Atlabachew M., Ferede E. (2023). The synthesis of decyl-o-phenanthrolinium organic salt and a study of the impact of anion type on *in vitro* antibacterial activities. Sci. Afr..

[24] Friedrich C.L., Moyles D., Beveridge T.J., Hancock R.E.W. (2020). Antibacterial Action of Structurally Diverse Cationic Peptides on Gram-Positive Bacteria. Antimicrob. Agents Chemother..

[25] Fletcher J.T., Sobczyk J.M., Gwazdacz S.C., Blanck A.J. (2018). Antimicrobial 1,3,4-trisubstituted-1,2,3-triazolium salts. Bioorg. Med. Chem. Lett..

[26] Da Costa D., Exbrayat-Héritier C., Rambaud B., Megy S., Terreux R., Verrier B., Primard C. (2021). Surface charge modulation of rifampicin-loaded PLA nanoparticles to improve antibiotic delivery in *Staphylococcus aureus* biofilms. J. Nanobiotechnol..

[27] Hasan N., Cao J., Lee J., Hlaing S.P., Oshi M.A., Naeem M., Ki M.H., Lee B.L., Jung Y., Yoo J.W. (2019). Bacteria-Targeted Clindamycin Loaded Polymeric Nanoparticles: Effect of Surface Charge on Nanoparticle Adhesion to MRSA, Antibacterial Activity, and Wound Healing. Pharmaceutics.

[28] Romero-Urbina D.G., Lara H.H., Velázquez-Salazar J.J., Arellano-Jiménez M.J., Larios E., Srinivasan A., Lopez-Ribot J.L., Yacamán M.J. (2015). Ultrastructural changes in methicillin-resistant *Staphylococcus aureus* induced by positively charged silver nanoparticles. J. Nanotechnol..

[29] Lange A., Beier S., Huson D.H., Parusel R., Iglauer F., Frick J.S. (2018). Genome sequence of *Galleria mellonella* (greater wax moth). Genome Announc..

[30] Yang H.F., Pan A.J., Hu L.F., Liu Y.Y., Cheng J., Ye Y., Li J.B. (2017). *Galleria mellonella* as an in vivo model for assessing the efficacy of antimicrobial agents against Enterobacter cloacae infection. J. Microbiol. Immunol. Infect..

[31] Meesters K., Alemayehu T., Benou S., Buonsenso D., Decloedt E.H., Pillay-Fuentes Lorente V., Downes K.J., Allegaert K. (2023). Pharmacokinetics of Antimicrobials in Children with Emphasis on Challenges Faced by Low and Middle Income Countries, a Clinical Review. Antibiotics.

[32] Paleczny J., Brożyna M., Dudek-Wicher R., Dydak K., Oleksy-Wawrzyniak M., Madziała M., Bartoszewicz M., Junka A. (2022). The Medium Composition Impacts *Staphylococcus aureus* Biofilm Formation and Susceptibility to Antibiotics Applied in the Treatment of Bone Infections. Int. J. Mol. Sci..

[33] Zhao R., Liang H., Clarke E., Jackson C., Xue M. (2016). Inflammation in Chronic Wounds. Int. J. Mol. Sci..

[34] Dydak K., Junka A., Dydak A., Brożyna M., Paleczny J., Fijalkowski K., Kubielas G., Aniołek O., Bartoszewicz M. (2021). In Vitro Efficacy of Bacterial Cellulose Dressings Chemisorbed with Antiseptics against Biofilm Formed by Pathogens Isolated from Chronic Wounds. Int. J. Mol. Sci..

